# Physiological pulmonary assessments in the management of bilateral diaphragmatic eventration

**DOI:** 10.1515/crpm-2021-0092

**Published:** 2022-06-27

**Authors:** Fahad Arattu Thodika, Emma E. Williams, Theodore Dassios, John Adu, Mahesh Nanjundappa, Christopher Harris, Anne Greenough

**Affiliations:** Department of Women and Children’s Health, School of Life Course Sciences, Faculty of Life Science and Medicine, King’s College London, London, UK; Neonatal Intensive Care Centre, King’s College Hospital NHS Foundation Trust, London, UK; Department of Paediatric Radiology, King’s College Hospital NHS Foundation Trust, London, UK; King’s College London, London, UK; NIHR Biomedical Research Centre based at Guy’s and St Thomas’, NHS Foundation Trust and King’s College London, London, UK

**Keywords:** diaphragm, electromyography, eventration, infant, physiological measurement

## Abstract

**Objectives:**

To describe the importance of comprehensive assessment to determine the underlying diagnosis and the role of physiological pulmonary measurements in the management of congenital bilateral eventration of the diaphragm.

**Case presentation:**

A female infant born at 34 weeks gestation required intubation and ventilation at birth. Chest radiographical imaging revealed bilateral density to the bases of both lung fields with raised hemi-diaphragms. Ultrasound imaging showed focal diaphragmatic eventration with bulging of the dome of the liver into the right and left hemithoraces. Assessment of the electrical activity of the diaphragm during a spontaneous breathing trial demonstrated a mean amplitude consistent with that of ventilated infants of the same gestational age with intact diaphragms. Hence she was extubated which was successful. Chest radiographic thoracic area measured post extubation was 1,654 mm^2^, equivalent to that of a term infant with severe congenital diaphragmatic hernia. As the electrical activity of the diaphragm was normal this suggests replacement of the diaphragmatic muscle tissue with fibrous bands was likely to be only partial, and hence why extubation was successful. She had other abnormalities presenting in the neonatal period including dermal melancytosis, central hypotonia, hyperinsulinism and poor feeding. The infant underwent extensive investigation which revealed a KMT2D gene mutation associated with Kabuki syndrome.

**Conclusions:**

Physiological pulmonary measurements may add clinical management in bilateratal diaphragmatic eventration.

## Introduction

Eventration of the diaphragm occurs unilaterally in 1 in 10,000 live births and bilateral eventration is a more rare event. Characterised by abnormal muscularization of the diaphragm, this congenital abnormality may result in impaired diaphragmatic motion and contractility. Diaphragmatic eventration may be isolated [1] or diagnosed in association with other congenital conditions (Table 1). Infants may present in the newborn period with respiratory distress soon after birth requiring mechanical ventilation and subsequent failure to extubate. Chest radiographs can reveal unilateral or bilateral density at the lung bases with raised hemi-diaphragm on the affected side, of which there are several differential diagnoses (Table 2). Diagnosis may, however, be made as late; in adulthood asymptomatic cases are incidentally diagnosed on radiographic imaging. Therapeutic options range from conservative management to invasive surgery depending upon the clinical severity. We report a case of bilateral eventration to demonstrate the importance of extensive investigation to determine the underlying diagnosis and the use of non-inasive physiological assessments, such as electromyography to provide adjunctive knowledge to clinicians and thus inform decision making and management.

**Table 1:** Conditions associated with congenital diaphragmatic eventration.

Congenital myotonic dystrophy
Cytomegalovirus
Kabuki syndrome
Fryns syndrome
Jarcho–Levin syndrome
Poland syndrome
Beckwith Wiedemann syndrome

**Table 2:** Differential diagnoses.

Pleural effusion
Pneumonia
Bronchogenic cyst
Congenital pulmonary airway malformation, CPAM
Congenital diaphragmatic hernia, CDH

## Case presentation

A female infant was born at 34 weeks of completed gestational age with a birth weight of 2,820 g. A complete course of antenatal corticosteroids had been given and routine antenatal imaging showed polyhydramnios and a large for date fetus. There was history of maternal gestational diabetes and pre-eclampsia.

**Figure 1:**
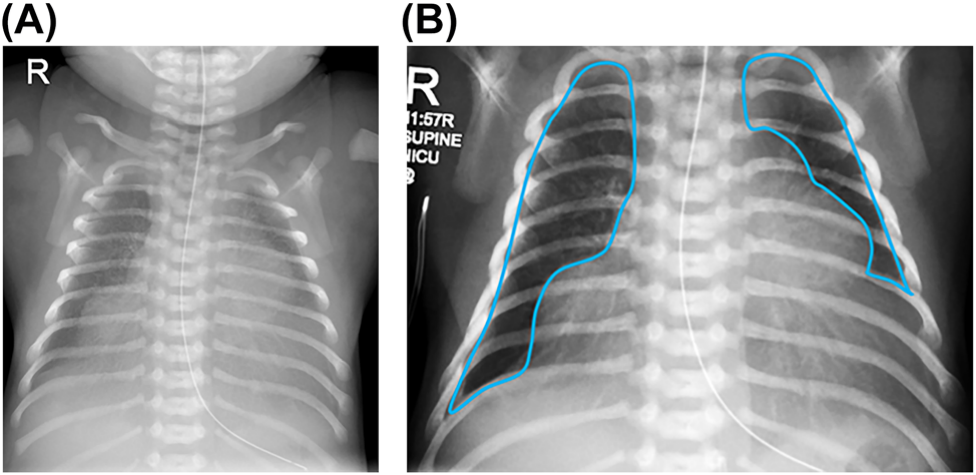
Plain chest radiograph (A) with definition of chest radiographic thoracic area (CRTA) showing bilateral diaphragmatic eventration (B).

**Figure 2:**
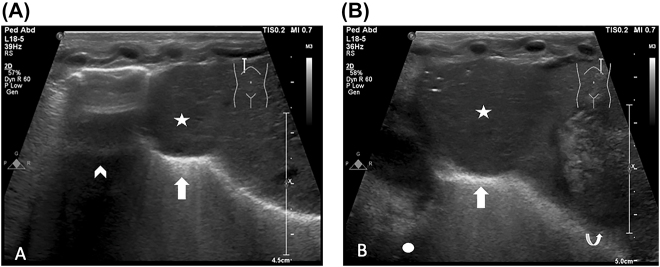
Selected longitudinal (sagittal) ultrasound images of right (A) and left (B) hemithoraces, demonstrating focal diaphragmatic eventration (arrows) with bulging of the dome of the liver into the eventration (stars). The right lower lobe of the lung (arrowhead), the heart (circle) and the stomach (curved arrow) are also visualised.

The infant was delivered by caesarean section in good condition with APGAR scores of 61, 95 and 1,010, but in view of respiratory distress she was given surfactant via less invasive administration 25 min after birth. She was then transferred to the neonatal intensive care unit on continuous positive airway pressure (CPAP) in 40% oxygen. At 4 h after birth in view of on-going respiratory distress and an increasing oxygen requirement up to 60% she was intubated and mechanically ventilated. A chest radiograph revealed evidence of possible bilateral eventration of the diaphragms (Figure 1). Diaphragm ultrasound examination (Figure 2) confirmed this suspicion. An echocardiogram (ECHO) performed at that time revealed mild hypoplasia of the isthmus, but a repeat ECHO demonstrated a structurally normal heart. After undergoing a spontaneous breathing trial and a period of non-invasive assessment of the electrical activity of the diaphgram, she was successfully extubated after 36 h of invasive ventilatory support. Futhermore, she did not require post-extubation non-invasive respiratory support and remained self-ventilating in air until discharge. As she did not require prolonged periods of invasive respiratory support, she was felt not to be a candidate for surgical intervention in the acute neonatal period.

During her neonatal course she was hypoglycaemic on admission to the neonatal unit with a blood glucose of 0.4 mmol/L and required a maximum glucose concentration of 20%. She was subsequently diagnosed with hyperinsulinism and commenced diazoxide. She had poor feeding despite normal videofluroscopy and ear, nose throat (ENT) examination and went on to require feeding support via a nasogastric tube. On neurological assessment she exhibited central hypotonia with brisk reflexes and was noted to have extensive melanocytic naevi over the lateral aspect of both arms with additional patches on the sacrum. Histopathological examination of a punch biopsy demonstrated results consistent with dermal melanocytosis. She was born with two neonatal teeth which were removed postnatally, however she subsequently developed two fibrous lesions on the anterior alveolar ridge which were pedunculated and mobile, these were later removed at 1 month of age. Extensive neurological investigation was performed and revealed normal brain magnetic resonance imaging scan, with normal upper and lower limb electromyography and nerve conduction studies. Considering the differential diagnosis of spinal muscular atrophy, immunohistochemistry analysis was performed, which showed normal results. Ophthalmological assessment and a renal ultrasound scan was performed, both were normal. Postnatal genetic testing revealed a KMT2D gene mutation, associated with Kabuki syndrome.

She was discharged home on day 64 after birth with nasogastric feeding support and continued on diazoxide medication. She continues to have extensive input from occupational therapists, speech and language therapists and physiotherapy teams.

### Imaging and measurements

The chest radiographic thoracic area (CRTA) measured post extubation (day three after birth) was 1,654 mm^2^ (Figure 1), equivalent to that of a term infant with severe congenital diaphragmatic hernia [2]. Ultrasound imaging (USS) (Figure 2) showed focal diaphragmatic eventration with bulging of the dome of the liver into the right and left hemithoraces. A spontaneous breathing trial (SBT) was conducted prior to exubation and electromyography of the diaphgram was measured during this period. The mean amplitude of diaphragmatic electrical activity was 2.47 μV, similar in magnitude to that of ventilated infants of the same gestational age with intact diaphragms. She successfully passed the SBT and subsequent extubation.

The parents gave informed written consent for this report and ethical approval was given for measuring the diaphragm EMG during the SBT prior to extubation (REC reference: 20/SW/0,062).

## Discussion

We described the rare event of bilateral diaphragmatic eventration which was associated with Kabuki syndrome, normal electrical activity of the diaphragm and reduced chest radiographic thoracic area.

Kabuki syndrome is a rare genetic condition associated with two gene mutations: KMT2D in the majority of cases and KDM6A less frequently [3]. There are many features suggestive of the diagnosis including characteristic facial dysmorphism, infantile hypotonia, faltering postnatal growth and developmental delay [4]. More rarely Kabuki can be associated with hyperinsulinism, and hence if the diagnosis is suspected glucose levels in the newborn period should closely monitored [5]. Eventration of the diaphragm is infrequently described in this condition [6], and in this case was detected on routine chest radiographic imaging.

Embryologically, development of the diaphragm occurs from the pleuroperitoneal folds, septum transversum and the somites with muscularization regulated by muscle progenitors [7]. In the present case there was normal electrical activity of the diaphragm, suggesting replacement of the diaphragmatic muscle tissue with fibrous bands was likely to be only partial and hence why extubation was successful. The lungs of infants with diaphragmatic eventration may be structurally normal, yet lung volume measured radiographically by calculation of chest thoracic area, may be reduced due to sub-optimal or incomplete contraction of the diaphragm during inspiration, with subsequent compression of the developing lung by abdominal viscera [8].

Primary surgical repair in those with bilateral eventration presenting in infancy may be necessary in cases associated with ventilator dependence [9]. The available options for such treatment can be minimally invasive surgery via thoracoscopy or invasive surgery via open thoracotomy. Thorascopic plication is considered the first choice of repair for infants and children with eventration due to faster post-operative recovery time, reduced length of hospital stays and lack of recurrence of symptoms [10].

We have highlighted the role physiological assessments can have in clinical management and decision making and in this case allowing for prompt successful early extubation of an infant with bilateral eventration of the diaphgram. This report also emphasises the importance of extensive investigation of an infant with bilateral diaphragmatic eventration to determine the underlying cause.

## Take home messages of lessons learnt

– Kabuki syndrome is a rare genetic condition associated with bilateral eventration of the diaphragm.
– Non-invasive physiological measurements may aid in management of patients presenting in infancy.
– Serial assessment of chest radiographic thoracic area and electrical activity of the diaphragm could be useful when considering optimal timing for intervention.
